# Circulating miR-30a, miR-195 and let-7b Associated with Acute Myocardial Infarction

**DOI:** 10.1371/journal.pone.0050926

**Published:** 2012-12-07

**Authors:** Guangwen Long, Feng Wang, Quanlu Duan, Shenglan Yang, Fuqiong Chen, Wei Gong, Xu Yang, Yan Wang, Chen Chen, Dao Wen Wang

**Affiliations:** Department of Internal Medicine and the Institute of Hypertension, Tongji Hospital, Tongji Medical College of Huazhong University of Science and Technology, Wuhan, People's Republic of China; University of Barcelona, Spain

## Abstract

**Background:**

MicroRNAs (miRNAs) play key roles in diverse biological and pathological processes, including the regulation of proliferation, apoptosis, angiogenesis and cellular differentiation. Recently, circulating miRNAs have been reported as potential biomarkers for various pathologic conditions. This study investigated miR-30a, miR-195 and let-7b as potential of biomarker for acute myocardial infarction (AMI).

**Methods and Results:**

Plasma samples from 18 patients with AMI and 30 healthy adults were collected. Total RNA was extracted from plasma with TRIzol LS Reagent. MiRNA levels and plasma cardiac troponin I (cTnI) concentrations were measured by quantitative real-time PCR and ELISA assay, respectively. Results showed that circulating miR-30a in AMI patients was highly expressed at 4 h, 8 h and 12 h after onset of AMI, and miR-195 was highly expressed at 8 h and 12 h. However, let-7b was lower in AMI patients than in controls throughout the whole time points. Interestingly, in these patients, circulating miR-30a, miR-195 and let-7b all reached their expression peak at 8 h. By the receiver operating characteristic (ROC) curve analyses, these plasma miRNAs were of significant diagnostic value for AMI. The combined ROC analysis revealed the an AUC value of 0.93 with 94% sensitivity and 90% specificity at 8 h after onset, and an AUC value of 0.92 with 90% sensitivity and 90% specificity at 12 h after onset, in discriminating the AMI patients from healthy controls.

**Conclusions:**

Our results imply that the plasma concentration of miR-30a, miR-195 and let-7b can be potential indicators for AMI.

## Introduction

MicroRNAs (miRNAs) are endogenous and non-coding single-stranded RNA molecules of approximately 23 nucleotides in length [1], [2], [3], [4]. By post-transcriptional targeting of mRNA, miRNAs induce translational degradation or inhibition of their targets [5], [6], [7]. MiRNAs have been reported play key roles in diverse pathological and biological processes, including proliferation, apoptosis, cell differentiation, cardiovascular diseases, neurological disorders, and cancers [8], [9], [10]. The crucial roles of miRNAs in cardiovascular system are supported by the findings that depletion of the miRNA-processing enzyme Dicer lead to defects in vessel formation, angiogenesis and cardiac development [11], [12], [13], [14].

Acute myocardial infarction (AMI) is one of the most serious cardiovascular diseases [15]. An early and accurate diagnosis can guarantee immediate initiation of reperfusion therapy to potentially reduce the mortality rate. Recent studies suggested that circulating myocardial-derived miRNAs might be useful as potential biomarkers for infarction [16], [17], [18], [19].

Previous studies demonstrated that miR-30a was associated with hypertrophy [20] and that miR-195 was up-regulated during cardiac hypertrophy in mice [21]. Validated targets of miR-195 regulated apoptosis, proliferation and cell cycle [22]. Moreover, recent studies showed a pro-apoptotic role of miR-195 in cardiomyocytes [23]. It was shown that expression of let-7 g was down-regulated in myocardial-injury mode [24]. Meanwhile, thioredoxin 1 induced over-expression of let-7 family inhibited cardiac hypertrophy [25]. Recently, it was reported that AMI modulated miR-1, -133a/b, and -499-5p plasma levels in humans and mice [26]. These results suggested that miRNAs may have fundamental roles in myocardial diseases. However, the expression levels of circulating miR-30a, miR-195 and let-7b in AMI remained unknown. In this study, we assessed the hypothesis that circulating miR-30a, miR-195 and let-7b may be useful for identifying and evaluating AMI.

## Materials and Methods

### 1. Ethics Statement

This study was conducted according to the principles expressed in the Declaration of Helsinki. This study was supported by the Ethics Committee of Tongji Hospital.

After obtaining the written informed consents, 5 ml blood samples were obtained from 18 patients and 30 healthy adults at Tongji Hospital from October 2009 to May 2010.

### 2. Blood Samples

AMI was diagnosed based on combination of several criteria: 1) ischemic symptoms; 2) increased cardiac cTnI level; 3) creatine kinase-MB (CK-MB); 4) pathological Q wave; and 5) ST-segment elevation or depression [27]. Meanwhile, 30 healthy volunteers (with normal electrocardiogram and no history of cardiovascular diseases) were enrolled in this study. The blood samples of patients with AMI were obtained at 4 h (±30 min), 8 h (±30 min), 12 h (±30 min), 24 h (±30 min), 48 h (±30 min), 72 h (±30 min) and 1 w (±60 min) after the onset of symptoms. Plasma was isolated by centrifugation and was maintained at −80°C until RNA extraction.

### 3. Plasma cardiac troponin I determine

Plasma cTnI concentrations were measured by ELISA assay according to the manufacturer's protocol (Abnova, Taiwan, China).

### 4. RNA Extraction

Total RNA was extracted from plasma with TRIzol LS Reagent as described previously [28].

### 5. miRNA qRT-PCR

Two micrograms of total RNA were reverse-transcribed using Transcript First-strand cDNA Synthesis SuperMix (TransGen Biotech, Beijing, China) according to the manufacturer's protocol. Briefly, the 50 µL reactions were incubated for 60 min at 42°C, 10 min at 70°C, and then preserved at 4°C. qRT-PCR were performed using the Bulge-Loop™ miRNA qRT-PCR Detection Kit (Ribobio Co., Guangzhou, China) and *TransStart*™ Green qPCR SuperMix (TransGen Biotech, Beijing, China) according to the manufacturer's protocol with the Rotor-Gene 6000 system (Corbett Life Science, QIAGEN, Hilden, Germany). In short, the reactions were incubated at 95°C for 30 s, and followed by 40 cycles of 95°C for 30 s, 60°C for 20 s, 70°C for 1 s. The relative expression levels for each miRNA were calculated by the comparative CT method. To avoid possible differences in the amount of starting RNA, resultant miRNA levels were normalized to small nuclear RNA U6.

### 6. Data analysis and statistics

Relative miRNA expression level was calculated by 2^−ΔΔct^ method [29]. Independent-samples T test was used for two-group comparisons. Comparisons of parameters among ≥3 groups were analyzed by repeated measures ANOVA. For categorical variables, the Chi-Square test was used. MiRNAs and cTnI time course trends were analyzed by repeated-measures ANOVA. All tests were 2-sided and a significance level of P<0.05 (95% CI) was considered statistically significant.

The ability to distinguish AMI group from control group was characterized by the receiver operating characteristic (ROC) curve, and the area under the ROC curve (AUC) was calculated. A composite score (miRNA-score) was defined to represent the cumulative level of the three miRNAs (miR-30a, miR-195 and let-7b) in the AMI group. The miRNA-score of each sample was calculated as the sum of the inverted-normalized signals of the three miRNAs and adjusted by subtracting a constant (the minimal score), so that the range of scores started at 0 [30].

All statistical analyses were performed using the statistical software SPSS 13.0 (Statistical Package for the Social Sciences, Chicago, Ill) for Windows.

## Results

### 1. Characteristics of patients

Among 18 patients with AMI, 13 were males and 5 were females, aged between 31 and 72 years old (mean 55±11.4). All patients had a transmural AMI. Total cholesterol, triglyceride, HDL, LDL, white blood cell, systolic blood pressure, diastolic blood pressure, creatinine, history of diabetes and smoking status were recorded, respectively. There were no significant statistical differences between AMI group and healthy group (P>0.05). Details were shown in Table 1.

**Table 1:** Clinical characteristics of patients.

Characteristics	Total patients (n = 48)	AMI (n = 18)	healthy outpatient (n = 30)	P
**Age (years)**	52±12	55±11.4	50±12.3	0.32
**Male/female (n/n)**	30/18	13/5	17/13	0.28
**Current smoking, n (%)**	34 (70%)	14 (78%)	20 (67%)	0.41
**DM, n (%)**	5 (10%)	3 (16%)	2 (6%)	0.28
**Hypertension, n (%)**	18 (38%)	9 (50%)	9 (30%)	0.16
**Hyperlipidaemia, n (%)**	3 (6%)	2 (11%)	1 (3%)	0.28
**Fasting glucose (mmol/L)**	5.98±1.71	6.28±1.37	5.8±1.8	0.49
**SBP (mmHg)**	128.9±19.9	130±17.5	127±17	0.67
**DBP (mmHg)**	79.7±15.8	82±17	77±14	0.4
**TC (mmol/L)**	4.3±0.9	4.33±0.9	4.30±1.0	0.9
**TG (mmol/L**	1.5±1.0	1.46±0.64	1.59±1.4	0.79
**HDL (mmol/L)**	1.16±0.29	1.05±0.25	1.24±0.31	0.11
**LDL (mmol/L)**	2.5±0.74	2.64±0.76	2.4±0.74	0.49
WBC (*10^9^/L)	7.07±2.0	8.0±2.5	6.5±1.47	0.073
**Cr (umol/L)**	68.7±33.78	90±47	58±20	0.1

DM, diabetes mellitus; SBP, systolic blood pressure; DBP, diastolic blood pressure; TC, total cholesterol; TG, total triglyceride; HDL, high-density lipoprotein; LDL, low-densitlipoprotein; WBC, white blood cell; Cr, creatinine. P, comparison between patients with healthy adults.

### 2. miRNAs and cTnI plasma levels in AMI patients and healthy adults

**Figure 1:**
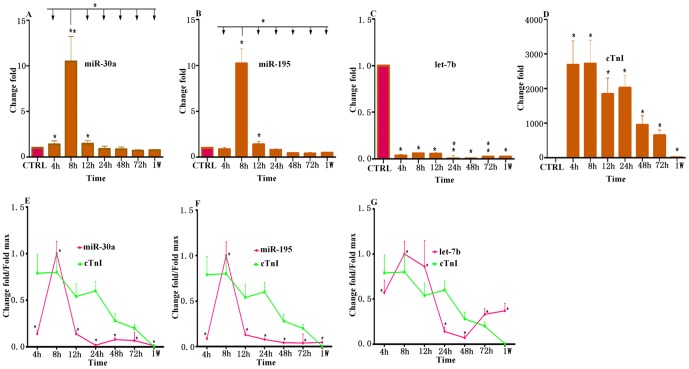
MiRNAs plasma levels in patients with AMI detected by real-time PCR assays. Plasma samples were collected at 4 h, 8 h, 12 h, 24 h, 48 h, 72 h and 1 w after the onset of symptoms. (A) The expression levels of miR-30a at different time points; (B) The expression levels of miR-195 at different time points; (C) The expression levels of let-7b at different time points; (D) Concentrations of cTnI at different time points. The results were reported as mean±SD. Values indicated fold changes of miRNAs vs. its level in the control group, arbitrarily set at 1 as indicated by CTRL. (E) Time courses of circulating miR-30a and cTnI from same plasma samples in patients with AMI and healthy control people; (F) Time courses of circulating miR-195 and cTnI from same plasma samples in patients with AMI and healthy control people; (G) Time courses of circulating let-7b and cTnI from same plasma samples in patients with AMI and healthy control people; The data were normalized to the peak level that each miRNA and cTnI achieved in each patient, and the time of the peak-fold increase vs. miRNAs and cTnI time courses were analyzed by repeated-measures ANOVA. Results were reported as mean+SD (*, p<0.05; **, p≤0.01).

Using qRT-PCR, we analyzed the expression levels of three miRNAs (miR-30a, miR-195 and let-7b) in AMI patients and healthy adults. We collected only few samples within 4 hours after onset, and there are no significant differences in plasma miRNA levels compared with controls (Fig. S1). Independent-samples T-test showed that levels of circulating miR-30a, miR-195 and let-7b were various in AMI patients and healthy adults after 4 hours (Table 2). Specifically, plasma miR-30a in patients with AMI exhibited a 1.4 (±0.37) fold, 10.48 (±2.75) fold and 1.45 (±0.34) fold increase at 4 h, 8 h and 12 h, respectively (Fig. 1A). Similarly, miR-195 exhibited a 10.2 (±1.61) fold and 1.4 (±0.3) fold increase in AMI group compared with control group at 8 h and 12 h, respectively (Fig. 1B). As shown in Figure 1, miR-30a and miR-195 plasma levels in AMI at each time point were compared. Interestingly, both miR-30a and miR-195 reached their circulating expression peak at 8 h compared with other time points. Oppositely, let-7b expression was down-regulated in AMI at all time points. Plasma levels of let-7b in AMI patients were 96%, 93%, 94%, 99%, 99.5%, 97.7% and 97.4% lower than in healthy control at 4 h, 8 h, 12 h, 24 h, 48 h, 72 h and 1 w, respectively (Fig. 1C).

**Table 2:** miR-30a, miR-195 and let-7b in human AMI patients and healthy adults.

		AMI (4 h)	AMI (8 h)	AMI (12 h)	AMI (24 h)	AMI (48 h)	AMI (72 h)	AMI (1 w)	healthy adult
**miR-30a**	**Δct±SD**	5.69±1.5	2.78±0.73	5.6±1.3	6.35±2.1	6.42±2.0	6.69±0.9	6.63±1.38	6.2±2.4
	**p value**	0.01	0.002	0.04					
	**AUC**	0.88	0.89	0.87					
**miR-195**	**Δct±SD**	−10.20±2.13	−13.73±2.17	−10.89±2.3	−10.07±1.3	−9.20±2.0	−9.15±2.3	−8.88±1.13	−10.37±0.65
	**p value**		0.02	0.04					
	**AUC**		0.89	0.88					
**let-7b**	**Δct±SD**	−6.4±2.0	−7.09±1.4	−6.9±2.3	−3.5±1.2	−5.67±2.9	−5.84±2.3	−6.5±1.3	−11.12±2.5
	**p value**	0.02	0.03	0.023	0.002	0.04	0.003	0.017	
	**AUC**	0.86	0.88	0.89	0.85	0.87	0.86	0.87	

Δct value of miR-195, miR-30a and let-7b in AMI groups and healthy adult is presented as an average group Δct±SD. Corresponding p values were calculated using the Independent-samples T test. And missing p values represent non significant Δct changes. AUC indicates the area under the receiver operating characteristic (ROC) curve for the discrimination between MI and control groups.

cTnI peak increase of 2720 (±680) fold was achieved at 8 h in AMI patients over control. cTnI levels of patients increased 2690 (±690) fold, 1840 (±460) fold, 2020 (±350) fold, 950 (±260) fold, 650 (±150) fold and 11.8 (±1.4) fold at 4 h, 12 h, 24 h, 48 h, 72 h and 1 w, respectively, compared with control (Fig. 1D).

### 3. Correlation of simultaneous plasma levels of miRNAs and cTnI in AMI patients

In 18 patients, miRNAs and cTnI were measured in the same plasma samples spontaneously. Meanwhile, miR-30a, miR-195, let-7b and cTnI time courses were analyzed by repeated-measures ANOVA in these patients. Interestingly, in these patients, plasma miR-30a, miR-195 and let-7b levels all reached their expression peak at 8 h, which is similar to the peak time of cTnI (Fig. 1E–1G).

### 4. Specifity and sensitivity of miRNAs for determination of AMI

We converted the expression level of miRNA into a single score to provide an improved signal to noise ratio. The score stood for the plasma levels of miRNA with P<0.0001 for the comparison between AMI and control group, and was calculated as described in the methods section.

**Figure 2:**
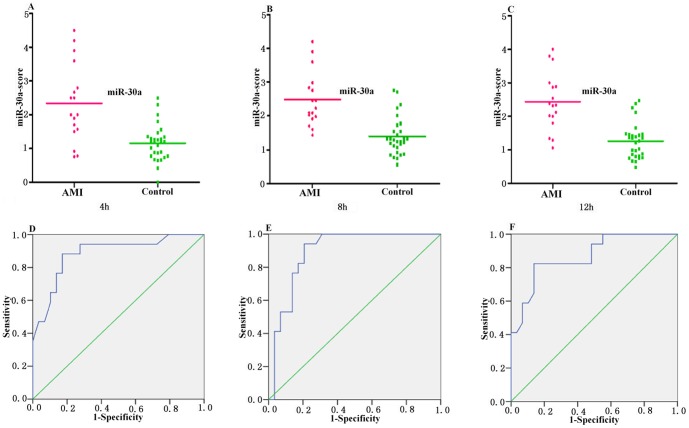
Circulating miR-30a expressions in AMI group and control group at 4 h, 8 h and 12 h. (A, B and C) The miR-30a-score was shown as median values in different groups; (D, E and F) ROC curve analyzed the diagnosis value of circulating miR-30a.

The median score of the miR-30a at 4 h, 8 h and 12 h is 2.41, 2.54 and 2.43 in the AMI group compared with 1.16, 1.59 and 1.26 in the control group (Fig. 2A–2C). The ability of the miRNA-score of miR-30a to discriminate the AMI group from the control group is demonstrated by the ROC curve with an AUC of 0.88, 0.89 and 0.87, respectively. By using the threshold score of 1.48, 1.65 and 1.47 above which patients were predicted to belong to the AMI group, we achieved a sensitivity of 88%, 88% and 82%, and a specificity of 83%, 80% and 80% for the identification of AMI patients, respectively (Fig. 2D–2F and Table 2).

**Figure 3:**
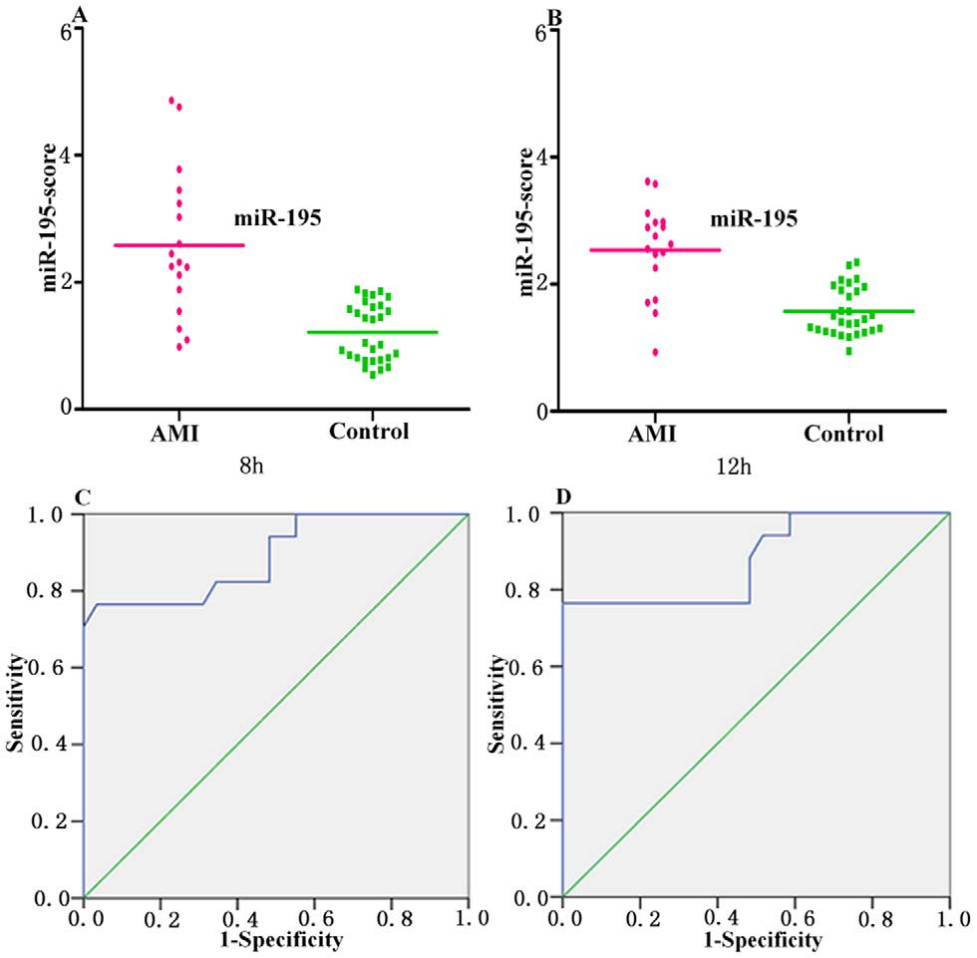
Circulating miR-195 expressions in AMI group and control group at 8 h and 12 h. (A and B) The miR-195-score was shown as median values in different groups; (C and D) ROC curve analyzed the diagnosis value of circulating miR-195.

The median score of miR-195 at 8 h and 12 h are 2.58 and 2.54 in the AMI group, compared with 1.2 and 1.21 in the control group (Fig. 3A and 3B), and AUCs are 0.89 and 0.88, respectively. Using the threshold score of 1.59 and 1.71, the sensitivity of miR-195 for the diagnosis of AMI in patients are 82% and 86%, and the specificity are 88% and 80% at 8 h and 12 h (Fig. 3C and 3D and Table 2).

**Figure 4:**
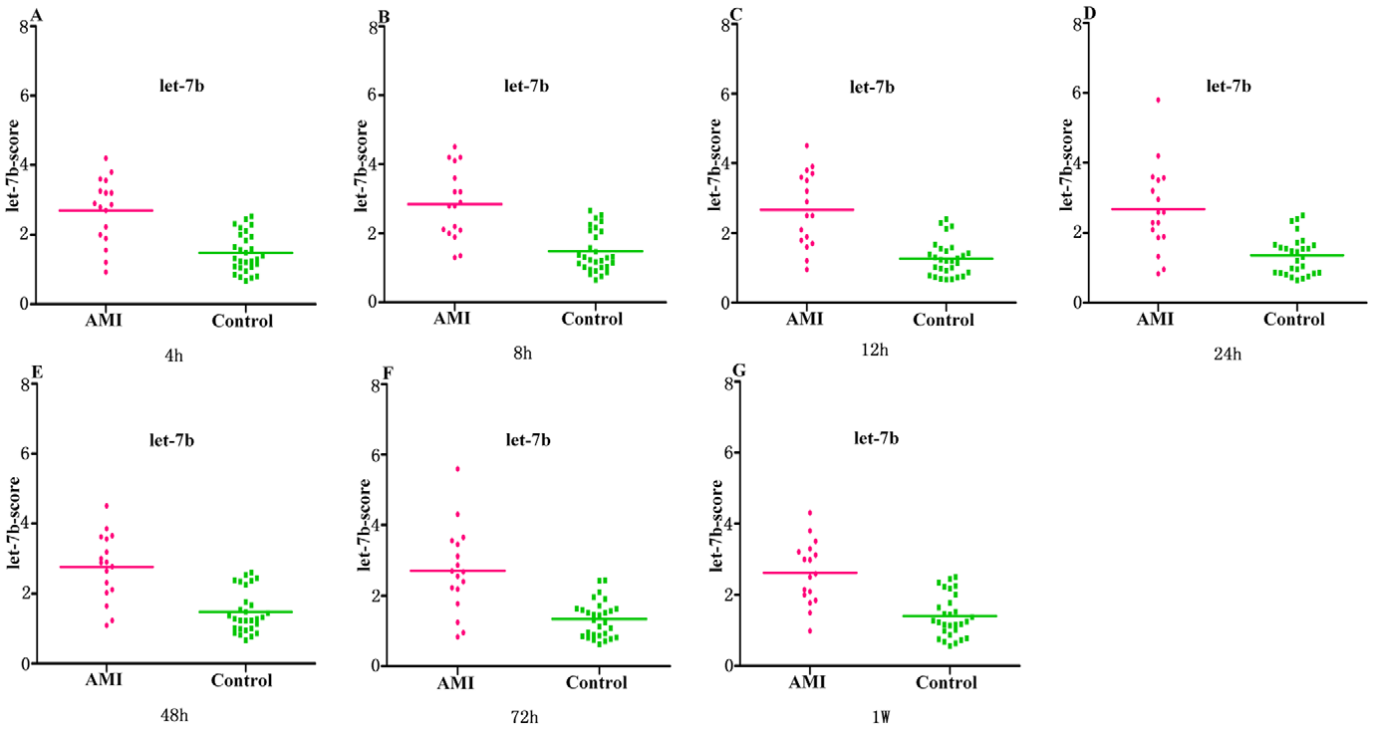
Circulating let-7b expressions in AMI group and control group at all different time points. (A–G) The let-7b-score was shown as median values in different groups.

**Figure 5:**
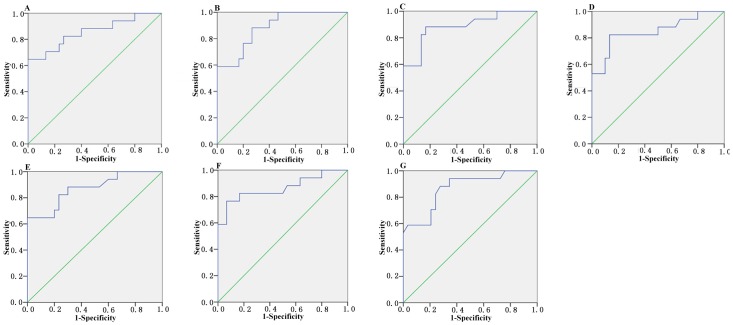
Circulating let-7b expressions in AMI group and control group at all different time points. (A–G) ROC curve analyzed the diagnosis value of circulating let-7b.

The median score of let-7b at 4 h, 8 h, 12 h, 24 h, 48 h, 72 h and 1 w are 2.7, 2.85, 2.67, 2.68, 2.77, 2.71 and 2.63 in the AMI group, compared with 1.46, 1.45, 1.26, 1.34, 1.46, 1.32 and 1.38 in the control group, respectively (Fig. 4A–4G). The ROC curve of let-7b showed moderate ability to distinguish between the AMI group and the healthy control group at 4 h, 8 h, 12 h, 24 h, 48 h, 72 h and 1 w, with an AUC value of 0.86, 0.88, 0.89, 0.85, 0.87, 0.86 and 0.87, respectively. The sensitivity of let-7b for the diagnosis of AMI in patients is 82%, 88%, 88%, 82%, 82%, 82% and 88%, and the specificity is 77%, 74%, 84%, 87%, 78%, 84%, 74% at 4 h, 8 h, 12 h, 24 h, 48 h, 72 h and 1 w, respectively (Fig. 5A–5G and Table 2).

**Figure 6:**
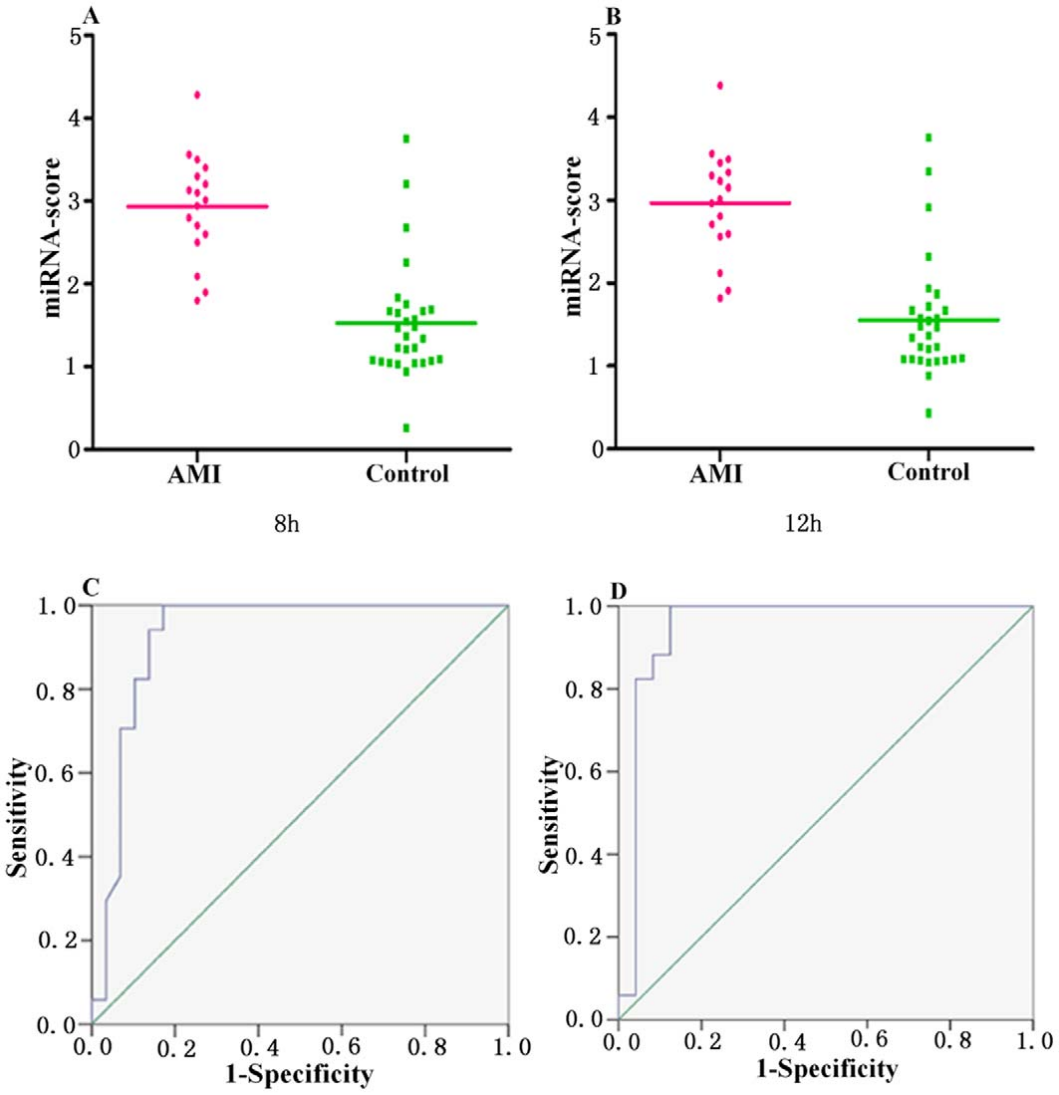
Discrimination between AMI and control group at 8 h and 12 h using the composite miRNA score (miRNA-score). (A and B) The composite miRNA-score was shown as median values in different groups; (C and D) ROC curve analyzed the diagnosis value of the composite miRNA-score.

When analyzed separately, each miRNA only showed moderate ability to distinguish the AMI group from the healthy control group, and none of them reached a sensitivity of 90% or a specificity of 90%. Since the detection of miRNA expression level may affected by both technical and biological variation, we combined the expression levels of the three circulating miRNAs into a single score, termed composite-miRNA-score, to provide an improved signal to noise ratio. Differently from the individual miRNA-score, the composite-miRNA-score represented the cumulative plasma levels of the three miRNAs (miR-30a, miR-195 and let-7b) with a strong differentiation (P<0.0001) for the comparison between AMI and controls, which was described in the methods section. The median score of composite-miRNA-score were 2.93 and 2.96 in AMI group and 1.53 and 1.56 in control group at 8 h and 12 h, respectively (Fig. 6A and 6B). The ability of the composite-miRNA-score to distinguish AMI group from control group was showed by the ROC curve with an AUC of 0.93 and 0.92. By using a threshold score of 1.815 and 2.025, above which patients were predicted to belong to the AMI group, a sensitivity of 94% and 90%, and a specificity of 90% and 90% were achieved for the identification of AMI patients, respectively (Fig. 6C and 6D).

## Discussion

Ischemic heart disease is the leading cause of human mortality and morbidity in the world, underscoring the need for innovative new therapies [31]. Accumulating evidences showed the importance of circulating miRNAs as stable blood-based biomarkers for cancers [32]. Moreover, recent studies indicated that some miRNAs that were selectively and/or highly expressed in AMI, for instance, miR-1, −133a, −133b, −208 and −499-5p, which were determined as biomarkers in myocardial injury [33], [34]. In this study, we reported the levels of circulating miR-30a, miR-195 and let-7b in human AMI, in comparison with the healthy adults. Results showed that miR-30a plasma levels in patients with AMI were up-regulated at 4 h, 8 h and 12 h, and circulating miR-195 expressions were up-regulated at 8 h and 12 h after the onset of AMI symptoms. Oppositely, let-7b was down-regulated in AMI at 4 h, 8 h, 12 h, 24 h, 48 h, 72 h and 1 w after the onset of AMI symptoms. The plasma concentrations of miR-30a, miR-195 and let-7b showed a good correlation with the plasma concentrations of cTnI, a classic marker of myocardial injury. Interestingly, in these patients, plasma miR-30a, miR-195 and let-7b levels reached their expression peak all at 8 h, which was similar to the peak time of cTnI. The ability of the three miRNAs-score to distinguish the AMI group from the control group was shown by the ROC curve with the AUC of 0.93 and 0.92 at 8 h and 12 h. By using a threshold score of 1.815 and 2.025, above which patients were predicted to belong to AMI group, we achieved a sensitivity of 94% and 90%, and a specificity of 90% and 90% for identification of AMI patients at 8 h and 12 h. Using the levels of these three miRNAs expression at 8 h and 12 h, we were able to define a score with a high sensitivity and specificity for the detection of AMI patients relative to matched control group. Thus, our results supported the hypothesis that miR-30a, miR-195 and let-7b may be useful for identifying the AMI.

To avoid possible bias from patient selection, subjects with similar age, gender, total triglyceride, white blood cell, total cholesterol, HDL, LDL, systolic blood pressure, diastolic blood pressure, creatinine, diabetes and smoking history were drawn into the present study. Statistical analyses further revealed these statuses did not influence miR-30a, miR-195 and let-7b levels in plasma. These data implied that miR-30a, miR-195 and let-7b may be potential specific biomarkers for AMI.

It should be noted that the consideration of circulating miR-30a, miR-195 and let-7b as a biomarker for AMI is from a relatively small sample size at present, and larger clinical studies should be required to establish the case.

MiRNAs regulate gene expression by modulating the translation of specific mRNAs. Some deregulated miRNAs that respond to AMI were reported to be associated with cell differentiation, hypoxia, inflammation, fibrosis and development [35]. Moreover, miRNAs may play important roles not only in the normal development of the cardiovascular system but also in cardiovascular diseases [36]. These results imply that miRNAs have critical roles in AMI pathophysiological processes. MiRNAs are endogenous regulators of gene expression, it is reasonable to hypothesize that miR-30a, miR-195 and let-7b can be involved in the regulation of cardiovascular function after AMI. Further experimental studies are necessary to explore their effects and mechanisms.

In summary, our study supplies insights into the levels of circulating miRNAs in patients with AMI.

## Supporting Information

Figure S1 **MiRNAs plasma levels in patients with AMI detected by real-time PCR assays within 4 hours after onset.** Plasma samples were collected at 1 h, 2 h and 3 h after the onset of symptoms. (A) The expression levels of miR-30a at different time points; (B) The expression levels of miR-195 at different time points; (C) The expression levels of let-7b at different time points. The data were normalized to the expression level of control groups, and analyzed by repeated-measures ANOVA. Results were reported as mean+SD (*, p<0.05; **, p≤0.01).
(DOC)
