# Supplementary material for: Circulating miR-30a, miR-195 and let-7b Associated with Acute Myocardial Infarction
Source: PLoS One. 2012 Dec 7;7(12):e50926. doi: 10.1371/journal.pone.0050926 (PMC3517576; doi:10.1371/journal.pone.0050926)
Supplement: Figure S1 — MiRNAs plasma levels in patients with AMI detected by real-time PCR assays within 4 hours after onset. Plasma samples were collected at 1 h, 2 h and 3 h after the onset of symptoms. (A) The expression levels of miR-30a at different time points; (B) The expression levels of miR-195 at different time points; (C) The expression levels of let-7b at different time points. The data were normalized to the expression level of control groups, and analyzed by repeated-measures ANOVA. Results were reported as mean+SD (*, p<0.05; **, p≤0.01). (DOC) [file pone.0050926.s001.doc]

**Supporting Information for PONE-D-12-21704**

**Circulating miR-30a, miR-195 and let-7b Associated with Acute Myocardial Infarction**

Guangwen Long^1*^, Feng Wang^1*^, Quanlu Duan^1^, Shenglan Yang^1^, Fuqiong Chen^1^, Wei Gong^1^, Xu Yang^1^, Yan Wang^1^, Chen Chen^1^ and Dao Wen Wang^1^

^1^Department of Internal Medicine and the Institute of Hypertension, Tongji Hospital, Tongji Medical College of Huazhong University of Science and Technology, Wuhan, People’s Republic of China

To whom correspondence should be addressed:

Chen Chen, MD, PhD

Department of Internal Medicine

Tongji Hospital, Tongji Medical College

Huazhong University of Science and Technology

1095# Jiefang Ave.,

Wuhan, 430030, P.R. China

Tel and Fax: (86-27)8366-3280

E-mail: [chenchen@tjh.tjmu.edu.cn](mailto:chenchen@tjh.tjmu.edu.cn)

**
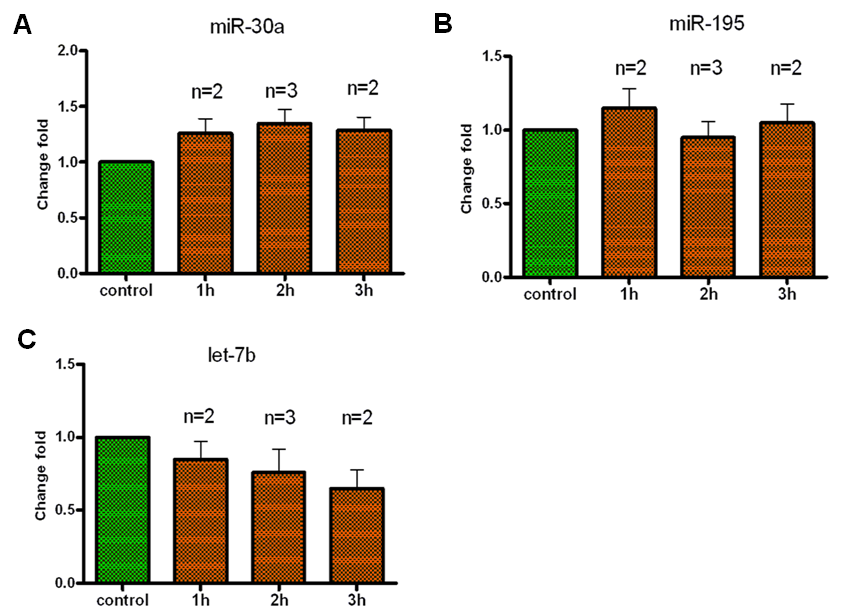
**

**Figure S1. MiRNAs plasma levels in patients with AMI detected by real-time PCR assays within 4 hours after onset.** Plasma samples were collected at 1h, 2h and 3h after the onset of symptoms. (A) The expression levels of miR-30a at different time points; (B) The expression levels of miR-195 at different time points; (C) The expression levels of let-7b at different time points. The data were normalized to the expression level of control groups, and analyzed by repeated-measures ANOVA. Results were reported as mean+SD (*, p<0.05; **, p≤0.01).
